# FamSeq: A Variant Calling Program for Family-Based Sequencing Data Using Graphics Processing Units

**DOI:** 10.1371/journal.pcbi.1003880

**Published:** 2014-10-30

**Authors:** Gang Peng, Yu Fan, Wenyi Wang

**Affiliations:** Department of Bioinformatics and Computational Biology, The University of Texas MD Anderson Cancer Center, Houston, Texas, United States of America; University of Canterbury, New Zealand

## Abstract

Various algorithms have been developed for variant calling using next-generation sequencing data, and various methods have been applied to reduce the associated false positive and false negative rates. Few variant calling programs, however, utilize the pedigree information when the family-based sequencing data are available. Here, we present a program, FamSeq, which reduces both false positive and false negative rates by incorporating the pedigree information from the Mendelian genetic model into variant calling. To accommodate variations in data complexity, FamSeq consists of four distinct implementations of the Mendelian genetic model: the Bayesian network algorithm, a graphics processing unit version of the Bayesian network algorithm, the Elston-Stewart algorithm and the Markov chain Monte Carlo algorithm. To make the software efficient and applicable to large families, we parallelized the Bayesian network algorithm that copes with pedigrees with inbreeding loops without losing calculation precision on an NVIDIA graphics processing unit. In order to compare the difference in the four methods, we applied FamSeq to pedigree sequencing data with family sizes that varied from 7 to 12. When there is no inbreeding loop in the pedigree, the Elston-Stewart algorithm gives analytical results in a short time. If there are inbreeding loops in the pedigree, we recommend the Bayesian network method, which provides exact answers. To improve the computing speed of the Bayesian network method, we parallelized the computation on a graphics processing unit. This allowed the Bayesian network method to process the whole genome sequencing data of a family of 12 individuals within two days, which was a 10-fold time reduction compared to the time required for this computation on a central processing unit.

This is a *PLOS Computational Biology* Software Article

## Introduction

Next-generation sequencing technologies have been employed routinely in detecting DNA variants and unveiling the cause of genetic diseases [1]. The broad application of next-generation sequencing technologies has led to an accompanying rapid development in variant calling algorithms and related software [2]–[5]. However, the variant calling error rate remains relatively high for rare variants [6], even though many new methods have been employed to improve variant calling, such as calling multiple samples together and borrowing information from the dbSNP database [7].

Roach et al. suggested using pedigree information to reduce the false positive rate of variant calling by removing all variants that do not conform to Mendelian transmission [8]. However, this method cannot control the false negative rate and cannot find any de novo mutations. Pedigree information has also been used to improve the accuracy for haplotype phasing in small families [9], [10]. Recent studies have shown that incorporating pedigree information into the variant calling reduces both false positive and false negative rates for family trios and extended families [11]–[14]. Peng et al. showed that in some HapMap families, incorporating pedigree information can reduce the false positive rates by 14–33% [12].

Several software packages have been implemented to incorporate pedigree information for variant calling. SAMtools [13] and DeNovoGear [14] can process family trios together. The Elston-Stewart algorithm was used in PolyMutt [11] to incorporate extended families. However, the Elston-Stewart algorithm requires either loop-cutting techniques, which will substantially increase the computing time and give approximate answers that are not always close to the exact results [15], or the use of the method proposed by Cannings et al. [16] that is hard to implement and has large memory requirements. Peng et al. proposed additional computational solutions for implementing the Mendelian genetic model in sequence variant calling [12]. The Bayesian network algorithm, in particular, provides exact results for a family pedigree with inbreeding loops. In order to allow for uncertainty in the minor allele frequency estimation, we also implemented a Markov chain Monte Carlo algorithm [17] to perform the family-based variant calling. To incorporate pedigree information into variant calling, we provide a program, FamSeq, that allows users to choose among the four following approaches, the Elston-Stewart algorithm, the Bayesian network algorithm, the graphics processing unit (GPU) version of the Bayesian network algorithm and the Markov chain Monte Carlo algorithm. FamSeq further improves the computational efficiency by using the GPU.

In whole genome sequencing, there are billions of loci with millions of candidate variant positions, so computing time is always a problem. We therefore sought to parallelize the Bayesian network algorithm in order to make the computing time feasible for analyzing a large set of whole genome sequencing data. GPUs were originally designed to accelerate the processing of graphics. As GPUs have become more programmable and have performed powerfully in parallel computing, they have been widely used in general-purpose applications, including those used in bioinformatics [18]–[20]. The Bayesian network algorithm contains many homogeneous tasks that can be accomplished by GPU parallel computing. Therefore, we implemented the parallel computing of the Bayesian network algorithm using the CUDA parallel computing platform on an NVIDIA GPU, which substantially increased the performance of that algorithm.

## Design and Implementation

### Design Overview

We developed a software package, FamSeq, which calls variants for family-based sequencing data. We used different methods to implement Mendelian transmission in FamSeq.

**Figure 1:**
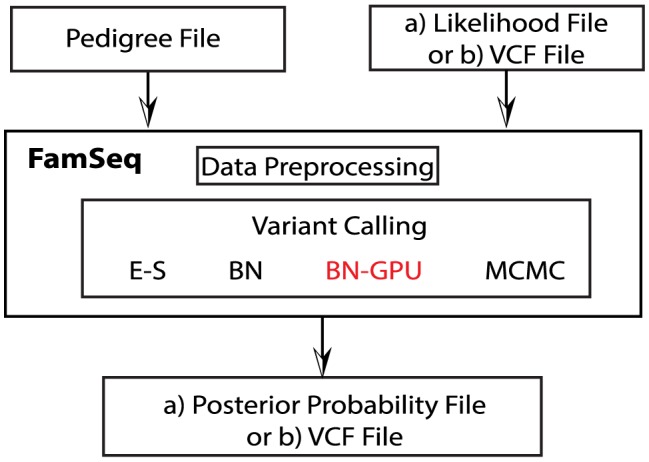
Workflow of FamSeq. We use a pedigree file and a file that includes the likelihood (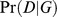) as the input to estimate the posterior probability (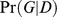) for each variant genotype. (E-S: Elston-Stewart algorithm; BN: Bayesian network method; BN-GPU: The computer needs a GPU card installed to run the GPU version of the Bayesian network method; MCMC: Markov chain Monte Carlo method; VCF: variant call format.)

**Figure 2:**
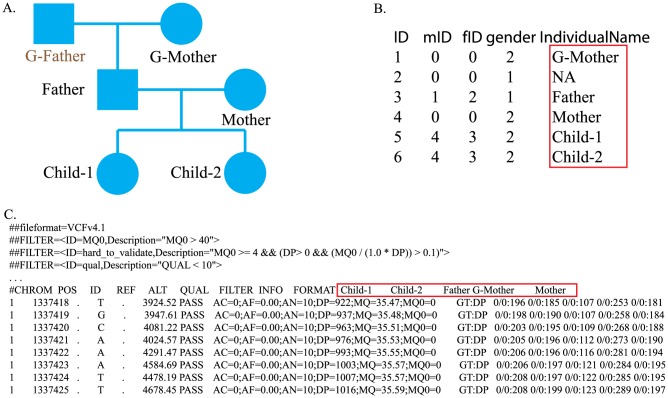
Illustration of input files. A.) Pedigree structure. B.) Pedigree structure file storing the pedigree structure shown in Fig. 2A. From the left-most column to the right-most column, the data are ID, mID (mother ID), fID (father ID), gender and sample name. C.) Part of VCF file. From the VCF file, we can find that the genome of the grandfather (G-Father) was not sequenced. We add his information to the pedigree structure file to avoid ambiguity. For example, if we include only one parent of two siblings in the pedigree structure file, it will be unclear whether they are full or half siblings. The sample name in the pedigree structure file should be the same as the sample name in the VCF file. When the actual genome was not sequenced, we set the corresponding sample name as NA in the pedigree structure file.

As outlined in the workflow of FamSeq (Fig. 1), two files are required as data input: a pedigree structure file and a file containing the genotype likelihood 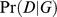, where *D* denotes the raw sequencing measurements, i.e., read counts, read quality and mapping quality, and *G* denotes the genotype of the individual. The pedigree file stores the individual identification (ID), parents' IDs, and gender and sample name, as is used to denote samples in the likelihood data file (Fig. 2). FamSeq accepts likelihood data files in two formats: a variant call format (VCF) [21] and a likelihood-only format (see description in our software manual). We introduced the likelihood-only format to allow for data generated from other sequencing platforms, with the requirement that the likelihood for each genotype is available.

FamSeq takes as input the two data files and settings of parameters (details on allele frequency and de novo mutation rate are shown hereafter). A data preprocessing feature of FamSeq will check whether there are any errors in the two input files. After that, FamSeq will implement the method the user chooses to call the variants.

FamSeq creates a new file as the output file, which follows the format of the input file but adds additional columns, with results on the posterior probability and the genotype, calculated using both the individual-based method and the family-based method.

### Data Preprocessing

FamSeq first checks the pedigree file. FamSeq requires the input pedigree to be complete, which means that everyone listed in the pedigree should have both a father and a mother represented in the pedigree file, with the exception of the founders of the family (Fig. 2). Otherwise, if two siblings have only one parent's information in the pedigree, FamSeq cannot determine whether they are full siblings or half siblings. FamSeq also checks for any inconsistency in the pedigree file, such as the father being erroneously listed as female. FamSeq extracts likelihood information from the Phred-scaled likelihood (PL) section in a VCF file or directly from a likelihood-only file.

### Input of Allele and Genotype Frequency in the Population

We require 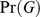, which is the probability of the genotype in a population. In FamSeq, we consider a bi-allelic model with reference (R) and alternative (A) alleles. Consequently, there are three kinds of genotypes in a diploid genome: RR, RA and AA. Without compromising the detection of true variants, we set the default value of the frequency of three genotypes in the population at 0.9985, 0.001 and 0.0005 if the variant is not represented in dbSNP. The dbSNP information should be provided by the input VCF file. For dbSNPs, the default value is set at 0.45, 0.1 and 0.45. Users can choose to set other values. When only the allele frequency is known, users can set genotype frequencies based on the Hardy-Weinberg equilibrium [22]. Based on findings from Peng et al., changes in the values of 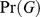 can affect the variant calling results of the founders, while its influence on offsprings in the family is small [12].

### Rate of De Novo Mutation

In FamSeq, we require the input of the de novo mutation rate by assigning a probability of *m* for each parental allele to mutate into the other allele in the germline [12]. In other words, when the two parents have homozygous reference genotypes, there is still a probability that their child has a genotype with an alternative allele. We added the de novo mutation rate in the calculation of transmission probabilities (described under Model Implementation).

The de novo mutation rate has been estimated to be around 1e−8 per base per generation [23]. When we analyzed real data, we found that the rates of false positives and false negatives were better controlled when a de novo mutation rate was set at 1e−7. Thus, we set a de novo mutation rate of 1e−7 as the default in FamSeq. Users can set the de novo mutation rate according to their requirements. In general, when the de novo mutation rate is set to a large value, the influence of pedigree-to-variant calling is small and the identification of more de novo mutations is allowed during variant calling.

Even though we allow for de novo mutations in our model, we still may over-correct the variant calling at some loci by following Mendelian inheritance principles when there are true de novo mutations. Therefore, we provide the following option to alleviate the over-correction: when the likelihood ratio for all individuals in the pedigree is larger than a user-specified cutoff and the genotypes do not follow Mendel's law, FamSeq will call variants using the individual-based method instead of the family-based method.

### Method Implementation

#### Markov chain Monte Carlo (MCMC) algorithm

We use the Gibbs sampler to derive the posterior probabilities for each genotype [17], [24]. During Gibbs sampling, the genotype of each individual in the family is updated, one at a time, based on the condition of all other family members' genotypes, the family configuration and the raw sequencing measurements. According to Mendelian segregation principles, the genotype of the individual does not depend on those of all family members, but only on the individual's parents, spouse and children. We can write the full conditionals as follows:
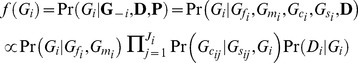
(1)where 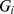 denotes the genotype for individual *i*, 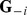 denotes the genotype for all family members, except individual *i*, **D** denotes the raw sequencing measurements, and **P** denotes the pedigree configuration. 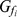, 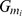, 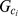 and 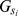 indicate the genotype of individual *i*'s father, mother, child and spouse. 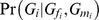 is the transmission probability, which shows how the parents' genotypes influence the child's genotype.

To avoid a local maximization problem in the Gibbs sampler, we also implemented a heated-Metropolis algorithm in MCMC, as proposed by Lin et al. [24]. In the heated-Metropolis algorithm, 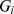 is sampled from a distribution of 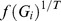 instead of 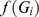. The sampled 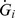 is accepted with the probability 
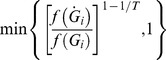
.

The accuracy of the MCMC algorithm depends on the number of iterations. As is shown in Biswas et al. [17], the MCMC approach requires tens of thousands of iterations to converge for a large pedigree; therefore, the computing time will also increase. By default, we set the number of iterations at 20,000*n*, where *n* is the pedigree size. Users can specify the number of iterations according to their needs.

#### Elston-Stewart algorithm

This algorithm splits the whole pedigree into anterior and posterior parts according to the individual of interest [25]. The anterior part relates to the parents of the individual, and the posterior part relates to the child/children of the individual. The probability of the anterior and posterior parts can be estimated recursively, such that the posterior genotype probability is calculated according to the probability of the anterior part and the posterior part. The Elston-Stewart algorithm is especially complex when there are inbreeding loops in the pedigree because then the pedigree cannot be directly split into anterior and posterior parts. There are two methods to solve this problem. First, we can cut the loops according to complex criteria and obtain an approximate result [15], [26]. Cannings et al. suggested using another method to obtain the analytical results [16]. However, their method has large memory requirements.

#### Bayesian network algorithm

By treating the entire pedigree as a directed acyclic graph (a Bayesian network), the genotype of sample *i* depends on the genotypes of only his/her parents [27]. We can write the posterior probability as
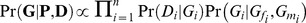
(2)The Bayesian network approach directly calculates the joint probabilities for all the combinations of genotypes of the whole family, and allows for analytic calculations for pedigrees with inbreeding loops. The Bayesian network approach is straightforward and easy to implement; however, the computing time increases exponentially when the pedigree size increases, so a supplementary approach is needed for a larger pedigree.

#### Bayesian network parallelization

For variant calling using whole genome sequencing data, there are billions of loci. After filtering by FamSeq, there are still millions of candidate variant positions remaining; thus, we propose to parallelize the Bayesian network algorithm in order to reduce the computing time and make this approach feasible in the DNA sequencing data analysis. In the Bayesian network method, we need to calculate the posterior probability for 3^n^ kinds of genotypes. This amounts to a large volume of homogeneous computing tasks that are suitable to parallel computing by GPUs.

Compared to central processing units (CPUs), GPUs have many advantages in parallel computing. A GPU usually has hundreds or thousands of core processors, while there are only several core processors for a CPU. Although the computing speed for each core processor of a GPU (about 1 GHz) is not as fast as that of a CPU (about 3 GHz), the total computing speed of a GPU is faster than that of a CPU. For a large amount of homogeneous computing tasks, we can assign one task to each GPU core to parallelize the computing.

**Figure 3:**
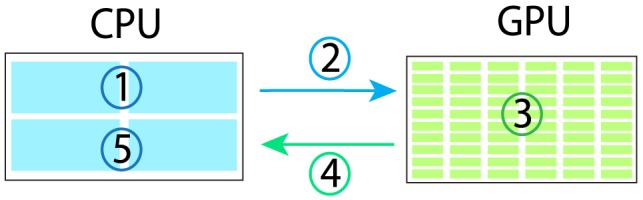
Illustration of GPU parallel computing in FamSeq. The program can be divided into two parts: a serial part and a parallel part. The serial part is processed in a CPU and the parallel part is processed in a GPU. The program: 1. Prepare the data for parallel computing in a CPU; 2. Copy the data from CPU memory to GPU memory; 3. Parallelize the 3^n^ jobs computing in the GPU, where n is the pedigree size; 4. Copy the results from GPU memory to CPU memory; and 5. Summarize the results in the CPU.

In FamSeq, we use CUDA (version 5.0 or later) to parallelize the Bayesian network algorithm on a GPU. CUDA is a parallel computing platform and programming model developed by NVIDIA. It can be implemented on many CUDA-enabled GPUs (https://developer.nvidia.com/cuda-gpus). CUDA provides many application programming interfaces that can be easily incorporated into C++ language. A brief illustration of GPU programming in FamSeq is shown in Fig. 3. For more details on GPU programming in C/C++, see the NVIDIA CUDA C Programming Guide (http://docs.nvidia.com/cuda/cuda-c-programming-guide/index.html).

## Results

We compared the computing time of the four different methods using real sequencing data with one million (1M) variants and a pedigree size that varied from 7 to 12. If there is no alternative allele at a position, this means that all individuals in the family have a homozygous reference genotype. If these positions are provided in the input VCF files, FamSeq will skip these positions and run joint calling on only the remaining potential variant positions. In order to estimate the actual computing time of FamSeq, we prepared a VCF file with 1M candidate variant positions as the input file. We tested the non-GPU version on a Linux server with Intel Xeon CPUs of 3.07 GHz. Only a single core of one CPU was used during testing. The GPU version was conducted on an NVIDIA Tesla M2090 with 512 cores of a 1.3 GHz GPU on a Linux server from Texas Advanced Computing Center (TACC). We used only one GPU during the comparison.

Table 1 shows the computing time for FamSeq based on using the CPU versus the GPU. The Elston-Stewart algorithm was the fastest among the four methods we used, and was the best choice when there were no inbreeding loops in the pedigree. The presence of inbreeding loops in the pedigree requires the use of loop cutting technology before calculating the probability of the anterior and posterior parts, which leads to algorithm complexity, increased computing time, and an approximation of the results. A big advantage of the Elston-Stewart algorithm is that the computing time increases almost linearly with increases in the pedigree size. When the pedigree size is large (greater than 12), the Elston-Stewart algorithm is almost the only computationally feasible method, especially for analyzing whole genome sequencing data.

**Table 1:** The total time (in seconds) needed for computation using FamSeq at one million positions.

Method	Loops	PU	Pedigree Size					
			7	8	9	10	11	12
E-S	N	CPU	13	12	15	16	22	34
MCMC^a^	N	CPU	100,920	129,030	160,170	177,740	240,650	296,600
	Y	CPU	117,460	233,490	289,720	362,630	432,760	496,750
BN	N	CPU	242	605	2,003	6,483	23,404	73,485
	N	GPU^b^	2,472 (150)	2,907 (169)	3,312 (239)	3,856 (397)	4,519 (946)	6,452 (2,717)
	Y	CPU	250	902	2,013	6,731	22,078	70,417
	Y	GPU^b^	2,548 (150)	2,986 (170)	3,123 (239)	3,602 (399)	4,396 (954)	6,605 (2,726)

PU: processing unit; E-S: Elston-Stewart algorithm; MCMC: Markov chain Monte Carlo algorithm; BN: Bayesian network algorithm; N: No, inbreeding loops are not considered; Y: Yes, inbreeding loops are considered.

a We called only 100,000 variants due to excessive running time for the MCMC algorithm. The time shown here is 10× the time required to call 100,000 variants.

b The time in parentheses is the GPU computing time.

Although the computing time for the Bayesian network algorithm increases exponentially when the family size increases, variant calling with this method can be completed in several hours for a pedigree with fewer than 10 individuals, based on whole genome sequencing data and assuming there are about 20 million candidate variant positions. When the pedigree size is small, the computing time difference between the Bayesian network algorithm and the Elston-Stewart algorithm is small. An advantage of the Bayesian network algorithm is that it can directly calculate posterior probabilities in pedigrees that have inbreeding loops. From Table 1, we show that the computing time for the Bayesian network algorithm is not affected by whether or not the pedigree has inbreeding loops.

We implemented the Bayesian network algorithm in both a CPU and GPU. Although we tried to increase the computing speed by parallelization at the GPU, the GPU version was slower than the CPU version when the pedigree size was less than 10. We found that transferring data between a CPU and GPU (steps 2 and 4 in Fig. 3) requires a lot of time and becomes a bottleneck for speed improvement with GPU parallelization. The number in the parentheses in Table 1 is the actual GPU computing time, which is only about one tenth of the total computing time. Since the time required to copy the data increases linearly and the computing time increases exponentially, the advantage in speed improvement for GPU parallelization becomes evident when the pedigree size is larger than 10. When the pedigree size was 12, the GPU version became 10 times faster than the CPU version, which made it feasible to call variants for the whole genome sequencing data in ∼36 hours as compared to more than 16 days for the CPU version. The actual improvement achieved from GPU computing will depend on its capacity, such as the total number of cores available in the GPU, which will vary from hundreds to thousands.

We ran FamSeq-GPU on a personal computer, a MacBook Pro with OS X 10.8.5, which has an NVIDIA GeForce GT 650M GPU containing 384 CUDA cores of up to 900 MHz. When the family size was 7, the corresponding GPU computing time was 360 s, which almost doubled the time needed by the TACC GPU server (Table 1), and the total computing time, including reading and writing between the CPU and GPU, was 3,060 s. We further observed that the GPU computing time increased to 1,970 s and the overall time increased to 7,300 s for a family size of 11. Our result shows if users do not have a professional computer server, they have an option of running FamSeq with parallel computing on a personal computer.

We also tested the computing time for our MCMC algorithm under the same settings (Table 1). Here, we set the total number of iterations at 20,000*n*, where *n* is the pedigree size. This option was the most time consuming and only provided approximate results. However, it can be used to analyze pedigrees with inbreeding loops and to incorporate uncertainty in the estimated alternative allele frequency, which is often not given as a set value, but as a value that follows a Beta distribution [14].

## Availability and Future Directions

FamSeq is a free software package under GNU license (GPL v3), which can be downloaded from our website: http://bioinformatics.mdanderson.org/main/FamSeq, or from SourceForge: http://sourceforge.net/projects/famseq/. According to feedback from current users, we will add to the output files an annotation of de novo mutations.

The present FamSeq provides the option of harnessing the power of GPUs that are manufactured by NVIDIA (CUDA parallel computing architecture). We plan to re-implement FamSeq using the Open Computing Language (OpenCL) so that FamSeq can be executed across heterogeneous platforms such as CPUs, GPUs, digital signal processors (DSPs), field-programmable gate arrays (FPGAs) and other processors.

## Supporting Information

Software S1 FamSeq software package.
(GZ) Click here for additional data file.
