# Supplementary material for: FamSeq: A Variant Calling Program for Family-Based Sequencing Data Using Graphics Processing Units
Source: PLoS Comput Biol. 2014 Oct 30;10(10):e1003880. doi: 10.1371/journal.pcbi.1003880 (PMC4214554; doi:10.1371/journal.pcbi.1003880)
Supplement: Software S1 — FamSeq software package. (GZ) [file pcbi.1003880.s001.gz › ./FamSeq/FamSeq_Manual.pdf]

Manual For FamSeq

Synopsis

FamSeq vcf -vcfFile input.vcf -pedFile input.ped -output output.vcf

FamSeq LK -lkFile lk.txt -pedFile input.ped -output output.txt

Commands and Options

vcf

FamSeq vcf [-method 1] [-mRate 1e-7] [-v] [-a] [-l] [-vcfFile] [-pedFile] [-output] [-LRC] [-genoProbN] [-genoProbK] [-genoProbXN] [-genoProbXK] [numBurnIn] [numRep]

Call variant when the input data is in a vcf file.

Options:	DT	Description
-method	*I*	The method used in variant calling. It is an integer. 1(default): Bayesian network. It works well when family size is less than seven. 2: Elston-Stewart algorithm. Use this method when family size is larger than 7 and the family has no loop. 3. MCMC.
-mRate	*F*	Mutation rate. It is a float. The default value is 1e-7.
-v		Only record the position at which the genotype is not RR in the output file. (R: reference allele, A: alternative allele).
-a		Record all the positions in the output file. If there is an indel at one position, FamSeq will write the same line in input vcf file to output vcf file. The number of lines in input vcf file and output vcf file are the same. If option -v is set, option -a will be discarded. If neither 'v' or 'a' is set, FamSeq will record all the positions except the indel positions.
-vcfFile	*STR*	The name of input vcf file. All the individuals must be in one vcf file.
-pedFile	*STR*	The name of ped file that store pedigree information. ***The pedigree should be a full family, which means that everyone in the family has two parents except for the founders of the family***. There are five columns in the ped file. The first column is individual id that should be larger than 0. The second and third column is mother's id and father's id. If the individual is the founder of the family, set the mother and father's id to 0. The forth column is gender. 1: male and 2: female. It will cause some errors at X chromosomes if the gender is not set correctly. The last column is individual name in vcf/likelihood only format file. If there is no

information of an individual in vcf/likelihood only format file, set the individual name to NA in the ped file. Example:

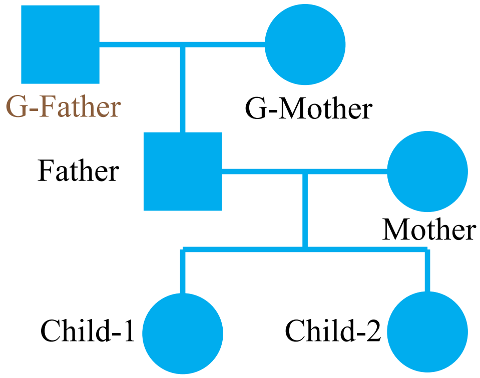

This is a family of six individuals. All individuals other than the grandfather were sequenced. The vcf file looks like the following:

```
##fileformat=VCFv4.1
##FILTER=<ID=MQ0,Description="MQ0 > 40">
##FILTER=<ID=hard_to_validate,Description="MQ0 >= 4 && (DP> 0 && (MQ0 / (1.0 * DP)) > 0.1)">
##FILTER=<ID=qual,Description="QUAL < 10">
...
#CHROM  POS      ID  REF  ALT  QUAL     FILTER  INFO                                      FORMAT  Child-1   Child-2   Father    G-Mother  Mother
1       1337418  .   T    .    3924.52  PASS    AC=0;AF=0.00;AN=10;DP=922;MQ=35.47;MQ0=0  GT:DP   0/0:196 0/0:185 0/0:107 0/0:253 0/0:181
1       1337419  .   G    .    3947.61  PASS    AC=0;AF=0.00;AN=10;DP=937;MQ=35.48;MQ0=0  GT:DP   0/0:198 0/0:190 0/0:107 0/0:258 0/0:184
1       1337420  .   C    .    4081.22  PASS    AC=0;AF=0.00;AN=10;DP=963;MQ=35.51;MQ0=0  GT:DP   0/0:203 0/0:195 0/0:109 0/0:268 0/0:188
1       1337421  .   A    .    4024.57  PASS    AC=0;AF=0.00;AN=10;DP=976;MQ=35.53;MQ0=0  GT:DP   0/0:205 0/0:196 0/0:112 0/0:273 0/0:190
1       1337422  .   A    .    4291.47  PASS    AC=0;AF=0.00;AN=10;DP=993;MQ=35.55;MQ0=0  GT:DP   0/0:206 0/0:196 0/0:116 0/0:281 0/0:194
1       1337423  .   A    .    4584.69  PASS    AC=0;AF=0.00;AN=10;DP=1003;MQ=35.57;MQ0=0 GT:DP   0/0:206 0/0:197 0/0:121 0/0:284 0/0:195
1       1337424  .   T    .    4478.19  PASS    AC=0;AF=0.00;AN=10;DP=1007;MQ=35.57;MQ0=0 GT:DP   0/0:208 0/0:197 0/0:122 0/0:285 0/0:195
1       1337425  .   T    .    4678.45  PASS    AC=0;AF=0.00;AN=10;DP=1016;MQ=35.59;MQ0=0 GT:DP   0/0:208 0/0:199 0/0:123 0/0:289 0/0:197
```

Then we construct the corresponding ped file. Make sure the individual name in the ped file is the same as in the vcf file. The grandfather should be included in ped file with individual name NA, even though there is no information about him in the vcf file.

ID	mID	fID	gender	IndividualName
1	0	0	2	G-Mother
2	0	0	1	NA
3	1	2	1	Father
4	0	0	2	Mother
5	4	3	2	Child-1
6	4	3	2	Child-2

```
##fileformat=VCFv4.1
##FILTER=<ID=MQ0,Description="MQ0 > 40">
##FILTER=<ID=hard_to_validate,Description="MQ0 >= 4 && (DP> 0 && (MQ0 / (1.0 * DP)) > 0.1)">
##FILTER=<ID=qual,Description="QUAL < 10">
...
#CHROM  POS     ID   REF   ALT   QUAL    FILTER  INFO    FORMAT  Child-1   Child-2   Father G-Mother   Mother
1   1337418  .   T   .   3924.52 PASS   AC=0;AF=0.00;AN=10;DP=922;MQ=35.47;MQ0=0    GT:DP  0/0:196 0/0:185 0/0:107 0/0:253 0/0:181
1   1337419  .   G   .   3947.61 PASS   AC=0;AF=0.00;AN=10;DP=937;MQ=35.48;MQ0=0    GT:DP  0/0:198 0/0:190 0/0:107 0/0:258 0/0:184
1   1337420  .   C   .   4081.22 PASS   AC=0;AF=0.00;AN=10;DP=963;MQ=35.51;MQ0=0    GT:DP  0/0:203 0/0:195 0/0:109 0/0:268 0/0:188
1   1337421  .   A   .   4024.57 PASS   AC=0;AF=0.00;AN=10;DP=976;MQ=35.53;MQ0=0    GT:DP  0/0:205 0/0:196 0/0:112 0/0:273 0/0:190
1   1337422  .   A   .   4291.47 PASS   AC=0;AF=0.00;AN=10;DP=993;MQ=35.55;MQ0=0    GT:DP  0/0:206 0/0:196 0/0:116 0/0:281 0/0:194
1   1337423  .   A   .   4584.69 PASS   AC=0;AF=0.00;AN=10;DP=1003;MQ=35.57;MQ0=0   GT:DP  0/0:206 0/0:197 0/0:121 0/0:284 0/0:195
1   1337424  .   T   .   4478.19 PASS   AC=0;AF=0.00;AN=10;DP=1007;MQ=35.57;MQ0=0   GT:DP  0/0:208 0/0:197 0/0:122 0/0:285 0/0:195
1   1337425  .   T   .   4678.45 PASS   AC=0;AF=0.00;AN=10;DP=1016;MQ=35.59;MQ0=0   GT:DP  0/0:208 0/0:199 0/0:123 0/0:289 0/0:197
```

	-output	*STR*	Output file name. If FamSeq calls a variant at a position, it will add two tags (FGT: genotype called by FamSeq and FPP: posterior probability estimated by FamSeq) at column FORMAT in vcf file.
	-LRC	*F*	A likelihood ratio cutoff. If likelihood (most likely genotype)/sum(likelihood of all genotypes) is less than the cutoff, we use pedigree information to improve variant calling. The default value is 1, we call all variant using pedigree information. Set it to 0 to only use single individual based method. Any values in between will determine whether FamSeq or single method is used for variant calling at a position.
	-genoProbN	*F F F*	Genotype probability of three kinds of genotype for autosome in population (Pr(G)) when this position is not in dbSNP. The default values are: 0.9985, 0.001 and 0.0005. The dbSNP position should be provided in column ‘ID’ in input vcf file.
	-genoProbK	*F F F*	Genotype probability of three kinds of genotype for autosome in population (Pr(G)) when the position is in dbSNP. The default values are: 0.45, 0.1 and 0.45.
	-genoProbXN	*F F*	Genotype probability of two kinds of genotype for chromosome X for male in population (Pr(G)) when the variant is not in dbSNP. The default values are: 0.999 and 0.001.
	-genoProbXK	*F F*	Genotype probability of two kinds of genotype for chromosome X for male in population (Pr(G)) when the variant is in dbSNP. The default values are: 0.5 and 0.5.
	-numBurnIn	*I*	Number of burn in when the user chooses the MCMC method. The default value is 1,000*n*, where *n* is the number of individuals in the pedigree.
	-numRep	*I*	Number of iteration times when the user chooses MCMC method. The default value is 20,000*n*.
LK	FamSeq LK [-method 1] [-mRate 1e-7] [-lkType n] [-v] [-a] [-l]		

[-lkFile] [-pedFile] [-output] [-LRC] [-genoProbN] [-genoProbK] [-genoProbXN] [-genoProbXK]

Call variants when the input data is in a likelihood only format file.

Options:	DT	Description
-lkFile:	*STR*	The name of input likelihood file. The first row is the individual name. The likelihood for each individual starts from the second row. Each column represents one individual. In each column, the likelihood for three kinds of genotype are sperated by comma.

```
Child1  Child2  Father  G-Mother  Mother
2.69e-06,0.209,0.0494    5.5e-12,0.00387,0.153       3.49e-08,0.0193,0.003       0.1,0.0985,1.38e-07        0.0239,0.115,9.18e-07
0.427,0.0331,1.24e-09    0.0032,0.272,9.12e-05       0.000354,0.395,0.00254      0.1,0.0985,1.38e-07        0.00466,0.332,0.000114
0.203,0.079,3.53e-08     0.000781,0.38,0.00102       4.75e-07,0.137,0.0786       8.72e-09,0.0479,0.183      6.33e-05,0.341,0.0091
0.376,0.0432,3.11e-09    0.505,0.00191,2.02e-13      0.268,0.0165,2.45e-10       0.392,0.000875,2.48e-14    0.0309,2.41e-06,6.6e-21
0.0493,0.0898,2.13e-07   1.19e-07,0.0399,0.00772     0.000791,0.0085,1.13e-08    0.011,0.209,1.21e-05       0.000354,0.395,0.00254
3.25e-07,0.107,0.0541    3.34e-08,0.00598,9.33e-05   0.18,0.0498,1e-08           2.69e-06,0.209,0.0494      0.000861,0.209,0.000154
```

-lkType	*STR*	The likelihood type. There are four types of likelihood: Normal (n), log10 scaled (log10), ln scaled (ln) and phred scaled (PS). The figure shown above is type n, without any scale.

DT: Data Type. *I*: integer. *F*: float value. *STR*: string.

Output

FamSeq creates a new file by adding three columns to the original input file as the output file: GPP, FPP and FGT. GPP is the posterior probability calculated by single individual based method and FPP is the posterior probability calculated by FamSeq. These probabilities are all Phred-scaled. FGT is the genotype called by FamSeq.

Version: 1.0.2
